# Impact of Responsibility Allocation Structures on Diagnostic Quality in AI-Assisted Diagnosis: Randomized Controlled Experiment

**DOI:** 10.2196/97588

**Published:** 2026-07-23

**Authors:** Tianya Liu, Ji Wu

**Affiliations:** 1 School of Business, Sun Yat-sen University, Guangzhou, Guangdong, 510275, China, 86 13113620362

**Keywords:** artificial intelligence, physician-AI collaboration, responsibility allocation, randomized controlled trial, diagnostic quality

## Abstract

**Background:**

AI is increasingly used to support clinical diagnosis, but the appropriate allocation of responsibility between clinicians and AI remains unclear. Different responsibility structures may influence how clinicians evaluate AI recommendations and revise their diagnostic judgments.

**Objective:**

This study aimed to examine how different physician-AI responsibility allocation structures affect diagnostic accuracy and confidence calibration during AI-assisted diagnosis.

**Methods:**

This individually randomized, 4-arm, parallel-group controlled experiment was conducted in a simulated clinical environment on the Credamo platform (Beijing Yishumofa Technology Co, Ltd). A total of 105 licensed physicians were randomly assigned to the dynamic responsibility, full responsibility, equal responsibility, or control group. Nine participants who failed the prespecified attention checks were excluded from the primary analysis, resulting in an analytic sample of 96 physicians. Participants completed 10 clinical vignette–based diagnostic tasks. The only between-group difference was the responsibility allocation structure. Primary outcomes were final diagnostic accuracy and confidence calibration; secondary outcomes included agreement rates and posttask subjective evaluations.

**Results:**

Responsibility allocation structures significantly modulated diagnostic quality. Compared to the control group, the full responsibility structure yielded no significant improvement in accuracy (mean 0.596, SD 0.152 vs 0.592, SD 0.169; mean difference 0.004, 95% CI −0.089 to 0.098; *P*=.93) or confidence calibration (mean 0.193, SD 0.134 vs 0.150, SD 0.145; mean difference 0.043, 95% CI −0.038 to 0.124; *P*=.29), while the equal responsibility structure showed suggestive evidence of lower diagnostic accuracy (mean 0.496, SD 0.185 vs 0.592, SD 0.169; mean difference −0.096, 95% CI −0.199 to 0.007; *P*=.07) and significantly poorer confidence calibration (mean 0.383, SD 0.175 vs 0.150, SD 0.145; mean difference 0.233, 95% CI 0.139 to 0.326; *P*<.001). Conversely, the dynamic responsibility structure demonstrated superior performance, significantly improving diagnostic accuracy (mean 0.717, SD 0.105 vs 0.592, SD 0.169; mean difference 0.125, 95% CI 0.043 to 0.207; *P*=.004) and reducing confidence calibration (mean 0.040, SD 0.084 vs 0.150, SD 0.105; mean difference −0.110, 95% CI −0.179 to −0.040; *P*=.003).

**Conclusion:**

The dynamic responsibility structure may enable health care organizations to use AI more fully and appropriately without compromising clinicians’ diagnostic performance, thereby improving the safety and quality of AI-assisted diagnosis.

## Introduction

The integration of AI into clinical workflows through decision support systems has transformed diagnostic and treatment pathways by providing real-time recommendations [1]. However, the rapid adoption of these technologies introduces significant challenges regarding accountability [2], particularly when an AI-assisted decision results in patient harm. Clinical decision-making in the age of AI is no longer a linear process; it is a complex interaction involving algorithm developers, health care organizations, and clinicians [3]. Consequently, responsibility can no longer be viewed as a simple causal chain assigned to a single actor but must be understood as a distributed responsibility chain spanning multiple phases and stakeholders [4].

This distributed structure has given rise to the responsibility gap, where the complexity and partial opacity of AI systems make it difficult to assign accountability under traditional legal and ethical mechanisms [5]. Beyond the legal vacuum, a critical behavioral concern is the phenomenon of responsibility diffusion [3]. Drawing on responsibility diffusion theory, the involvement of multiple actors may inadvertently diminish clinicians’ perceived personal accountability, potentially weakening their vigilance and critical evaluation before adopting AI-generated recommendations [6]. In the high-stakes environment of health care, where diagnostic errors directly threaten patient safety, this potential reduction in vigilance is particularly hazardous.

The tension between human judgment and automated advice is further complicated by existing liability structures. While AI-related adverse outcomes often stem from multistage technical and organizational failures, current responsibility arrangements frequently place the primary burden of liability on the individual clinician [7]. This concentration of liability may not only increase the perceived risk and uncertainty for physicians using AI but may also lead to a defensive decision-making posture that hinders effective human-AI collaboration [8].

Despite extensive conceptual debate regarding the responsibility gap, there remains a critical lack of causal evidence demonstrating how specific responsibility allocation structures influence clinicians’ decision-making performance and collaborative behaviors in AI-assisted diagnosis. To address this gap, we conducted a 4-arm, parallel-group randomized controlled experiment involving 96 verified hospital physicians recruited through a professional platform. By simulating a clinical environment with validated vignettes, we compared the effects of full, equal, and dynamic responsibility allocation structures against a control condition. The primary objective of this study is to identify actionable and traceable responsibility design mechanisms to enhance diagnostic accuracy and confidence calibration, thereby providing a robust evidence base for the design of safe and accountable human-AI workflows in health care systems.

## Methods

### Study Design and Setting

This study employed a preregistered [9], 4-arm, parallel-group randomized controlled trial to investigate the causal impact of different responsibility allocation structures on clinicians’ diagnostic performance and collaborative behaviors [10]. The experiment was conducted between December 2025 and January 2026 using a simulated clinical environment hosted on the Credamo platform (Beijing Yishumofa Technology Co, Ltd). This design allowed for the systematic comparison of 3 experimental responsibility allocation structures—full, equal, and dynamic responsibility—against a standard control condition during an AI-assisted diagnostic task.

### Participants and Recruitment

Participants were recruited through the Credamo platform and were required to be verified, licensed hospital clinicians capable of completing complex, vignette-based clinical decision-making tasks on digital devices. Upon providing electronic informed consent, participants were screened for eligibility and consistency. A total of 105 physicians were enrolled and randomly assigned to the 4 groups. Nine participants who failed the prespecified attention checks were excluded from the primary analysis, resulting in a final analytic sample of 96 physicians, with 24 participants in each group.

### Clinical Vignettes

Ten cases were randomly selected from the Chinese National Medical Licensing Examination question bank and adapted into clinical vignettes. Two clinical experts reviewed the case content and reference diagnoses and assessed the complexity and difficulty of each vignette; the final set comprised 5 easy and 5 difficult cases. A pilot test involving 20 licensed physicians was subsequently conducted to evaluate the clarity of the vignette descriptions, the comprehensibility of the experimental procedures, and the overall task difficulty; the experimental materials were then refined accordingly.

### Ethical Considerations

The study protocol was approved by the Ethics Committee of the School of Business, Sun Yat-sen University (approval number: BS20251227).

### Trial Registration

The study was preregistered on the AsPredicted platform (Wharton Credibility Lab) before data collection commenced. However, the trial was not prospectively registered in a clinical trial registry because we initially did not fully recognize that an online clinical vignette–based simulation involving licensed physicians also required prospective trial registration. The trial was subsequently registered retrospectively with the ISRCTN registry (ISRCTN16943519). The study design, primary outcomes, exclusion criteria, and planned analyses had been specified before data collection and remained unchanged throughout the study. No outcomes were selectively omitted, added, or modified on the basis of the study findings.

### Randomization

Within a system-prespecified vignette-based clinical decision-making task, participants were randomly assigned in a 1:1:1:1 ratio using computer-generated simple randomization to 1 of 4 responsibility allocation structures: a no-intervention control condition and 3 experimental conditions manipulating different responsibility allocation structures [11]. Allocation was implemented automatically by the platform back end, and the research team could neither foresee nor influence group assignment. As the intervention centered on the description and display of a responsibility allocation structure, participant blinding was not feasible; therefore, the trial used an open-label design. To minimize analytical bias, group labels were anonymized after data cleaning and coding, and statistical analyses were conducted by an analyst blinded to group identity [12].

### Intervention

**Figure 1.**
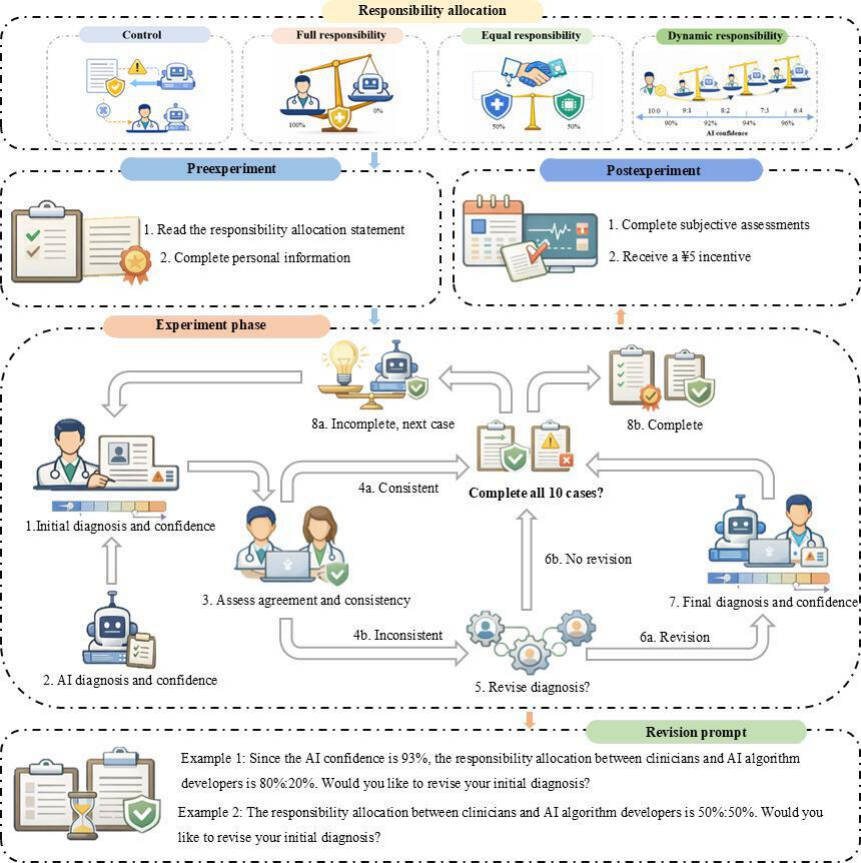
Experiment procedures. Currency conversion is based on the December 2025 average exchange rate (US $1=CN ¥7.0432).

The experimental workflow, illustrated in Figure 1, began with participants acknowledging a group-specific responsibility allocation statement that defined the accountability boundaries between the clinicians and the AI algorithm developers [13]. Participants then proceeded to the task interface where, after providing an initial diagnosis, they received diagnostic advice and rationale generated by ChatGPT (GPT-5.2). As the second component of the intervention, during each case response phase (before participants submitted their final diagnosis or revised their initial diagnosis), the system presented a group-specific adjustment prompt to reinforce the responsibility structure and guide final decision confirmation.

### Procedures

After entering the online experiment platform, participants first completed a personal information form and read the responsibility statement displayed by the system. They then completed diagnostic tasks for 10 clinical vignettes. For each case, participants made an initial diagnosis based on the case information and rated their confidence. Then, the system displayed the AI diagnostic conclusion and asked participants to rate agreement with the AI recommendation and to record human-AI outcome consistency. If the 2 conclusions were consistent, participants proceeded directly to the next case; otherwise, the system asked whether they wished to revise their initial diagnosis. After revising, participants rerated their confidence and submitted the final diagnosis for that case. After completing all cases, participants completed a posttask questionnaire assessing outcomes [14].

### Outcome Measures

Before the diagnostic tasks, participants reported their sociodemographic and professional characteristics, previous experience with AI, and trust in AI.

The postintervention outcomes included diagnostic quality, agreement, adjustment, and posttask subjective measures. Diagnostic quality comprised initial and final diagnostic accuracy, each coded dichotomously against the case-specific reference standard (1=correct diagnosis, 0=otherwise). We also calculated calibration for the final diagnosis to assess the concordance between confidence and actual correctness: confidence in the final diagnosis was rated on a 1 to 7 scale and linearly rescaled to a 0 to 1 range; accuracy was coded as 1 for correct and 0 for incorrect. Calibration was defined as the absolute difference between confidence and accuracy, with lower values indicating better calibration. Agreement captured whether participants endorsed key elements of the AI reasoning and was coded as a binary variable (1=agree, 0=otherwise). Adjustment reflected whether participants modified their diagnostic conclusion in response to the AI advice and was also coded dichotomously (1=adjusted, 0=otherwise). After completing the task, participants reported their subjective perceptions of collaborative efficacy, cognitive load, and credit attribution during the task using Likert-type scales, each scored from 1 to 7 (1=strongly disagree, 7=strongly agree).

### Data Analysis

Quantitative analyses were performed using Stata (version 15.1; StataCorp). We conducted group comparisons for primary and secondary outcomes, including diagnostic quality measures and posttask subjective scales. All tests were 2-sided. Results with *P*<.05 were considered to meet the conventional threshold for statistical significance, whereas those with *P*<.05-.10 were reported as suggestive evidence and were not interpreted as conclusive findings.

### Patient and Public Involvement

Patients and the public were not involved in the design, conduct, reporting, or dissemination plans of this research. The study involved licensed clinicians completing simulated vignette-based diagnostic tasks.

## Results

### Characteristics of Participants

**Figure 2.**
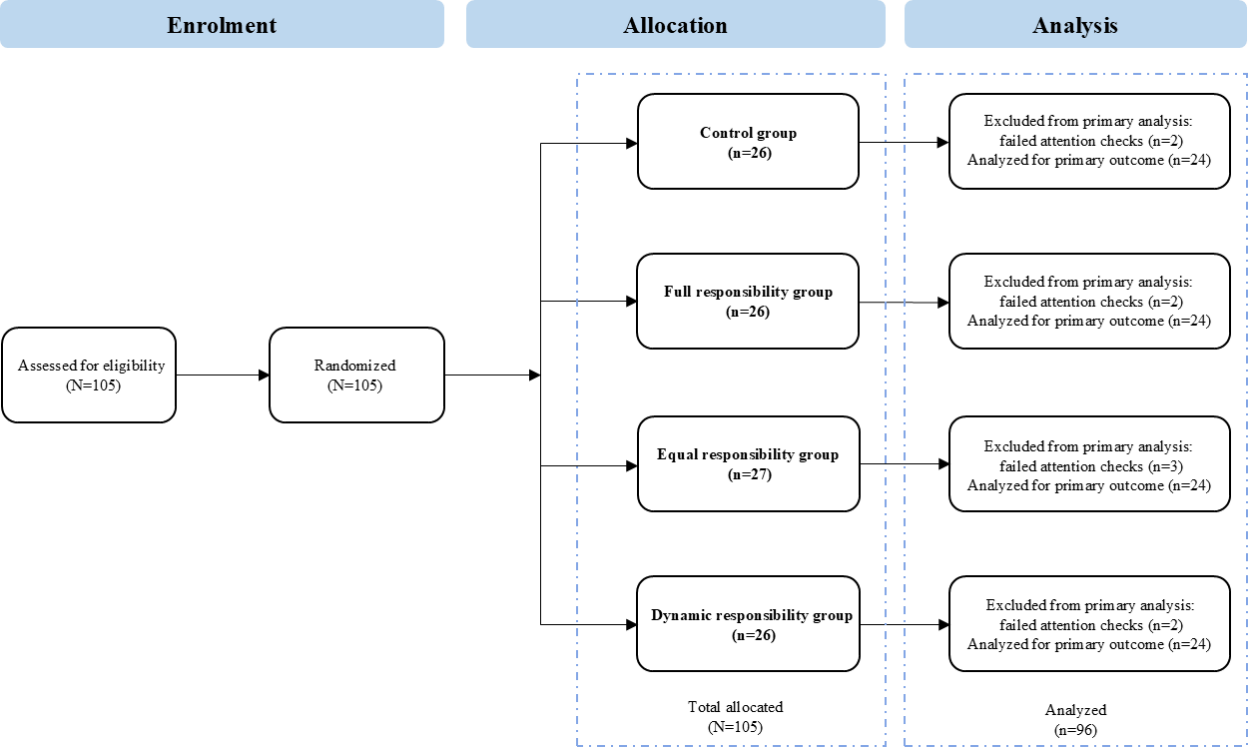
Flow diagram.

A total of 96 verified hospital physicians were successfully recruited and underwent 1:1:1:1 randomization into the 4 study arms (n=24 per group; Figure 2). Data from all 96 participants were included in the final primary analysis after passing internal consistency checks. The sample was balanced across genders (51/96, 53.1% female) and primarily composed of clinicians in early- to midcareer stages, with 87.5% (n=84) aged between 21 and 40 years. In the sample, 42.7% (n=41) held intermediate professional titles, and clinical experience was evenly distributed, with 51.1% (n=49) of the cohort having more than 6 years of practice. AI use experience was high, with 79.2% (n=76) of participants having used AI tools for 6 months or longer.

### Primary Outcomes

Responsibility allocation structures were significantly associated with overall diagnostic quality. The primary results, including point estimates and 95% CIs derived from the experimental data, are presented in Tables 1 and 2.

Compared to the control group, the full responsibility structure did not yield a statistically significant improvement in final diagnostic accuracy (mean 0.596, SD 0.152 vs mean 0.592, SD 0.169; difference 0.004, 95% CI –0.089 to 0.098; *P*=.93) or confidence calibration (mean 0.193, SD 0.134 vs mean 0.150, SD 0.145; difference 0.043, 95% CI –0.038 to 0.124; *P*=.29). The equal responsibility structure suggested a reduction in accuracy, but this was not statistically significant (mean 0.496, SD 0.185 vs mean 0.592, SD 0.169; difference –0.096, 95% CI –0.199 to 0.007; *P*=.07); furthermore, this structure resulted in significantly poorer confidence calibration compared to the control group (mean 0.383, SD 0.175 vs mean 0.150, SD 0.145; difference 0.233, 95% CI 0.139 to 0.326; *P*<.001), indicating a heightened mismatch between clinician confidence and objective accuracy.

In contrast, the dynamic responsibility structure demonstrated a robust advantage, significantly improving final diagnostic accuracy (mean 0.717, SD 0.105 vs mean 0.592, SD 0.169; difference 0.125, 95% CI 0.043 to 0.207; *P*=.004) and reducing confidence calibration (mean 0.040, SD 0.084 vs mean 0.150, SD 0.145; difference –0.110, 95% CI –0.179 to –0.040; *P*=.003). Pairwise comparisons further confirmed that the dynamic structure significantly outperformed both the full (accuracy *P=*.003; calibration *P<*.001) and equal responsibility models (accuracy *P*<.001; calibration *P*<.001), suggesting that embedding responsibility cues at points of human-AI disagreement promotes superior diagnostic verification.

**Table 1.** Diagnostic accuracy comparisons across responsibility allocation structures.

Comparison	Group mean (SD)	*t* test (*df*)	*P* value
Full responsibility vs control	0.596 (0.152) vs 0.592 (0.169)	0.09 (45.47)	.93
Equal responsibility vs control	0.496 (0.185) vs 0.592 (0.169)	–1.87 (45.63)	.07
Dynamic responsibility vs control	0.717 (0.105) vs 0.592 (0.169)	3.08 (38.42)	.004
Full vs equal responsibility	0.596 (0.152) vs 0.496 (0.185)	2.05 (44.28)	.047
Full vs dynamic responsibility	0.596 (0.152) vs 0.717 (0.105)	–3.21 (40.91)	.003
Equal vs dynamic responsibility	0.496 (0.185) vs 0.717 (0.105)	–5.08 (36.38)	<.001

**Table 2.** Diagnostic calibration comparisons across responsibility allocation structures. Note: Lower confidence calibration values indicate better calibration.

Comparison	Group mean (SD)	*t* test (*df*)	*P* value
Full responsibility vs control	0.193 (0.134) vs 0.150 (0.145)	1.08 (45.71)	.29
Equal responsibility vs control	0.383 (0.175) vs 0.150 (0.145)	5.01 (44.40	<.001
Dynamic responsibility vs control	0.040 (0.084) vs 0.150 (0.145)	–3.20 (36.94)	.003
Full vs equal responsibility	0.193 (0.134) vs 0.383 (0.175)	–4.21 (42.97)	<.001
Full vs dynamic responsibility	0.193 (0.134) vs 0.040 (0.084)	4.75 (38.76)	<.001
Equal vs dynamic responsibility	0.383 (0.175) vs 0.040 (0.084)	8.62 (33.05)	<.001

### Secondary Outcomes

**Figure 3.**
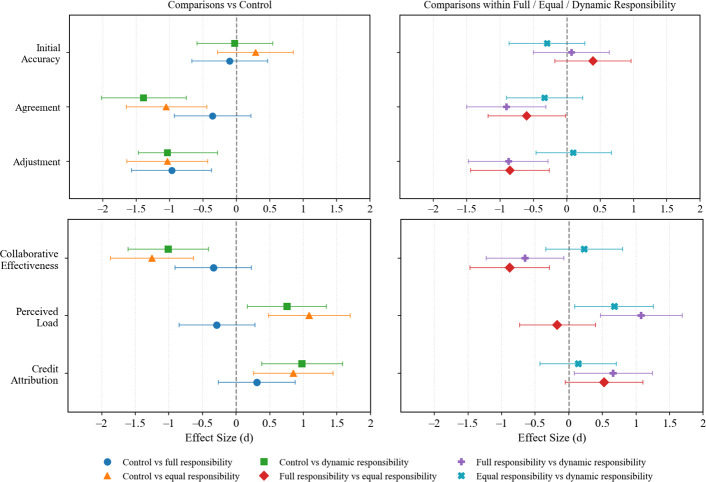
Secondary results.

Secondary outcomes are presented in Figure 3. For initial accuracy, no statistically significant differences were observed across responsibility allocation structures, indicating that the responsibility structures did not affect initial judgments. Compared with the control condition and the full responsibility structure, both the equal responsibility and dynamic responsibility structures showed higher levels of agreement and adjustment, although the difference between these 2 structures was not significant.

Regarding subjective evaluations, the equal responsibility and dynamic responsibility structures were associated with lower cognitive load, higher collaborative effectiveness, and lower credit attribution, suggesting that under these 2 responsibility allocation structures, clinicians were less likely to attribute diagnostic contributions entirely to themselves while experiencing a lighter task burden and a more favorable sense of collaboration.

## Discussion

### Principal Findings

The primary objective of this randomized controlled experiment was to evaluate how various responsibility allocation structures influence the diagnostic performance of clinicians when assisted by AI. Our findings indicate that responsibility structures may play an important role in shaping diagnostic quality. The principal finding is that a dynamic responsibility structure, in which accountability cues are specifically triggered during human-AI disagreement, significantly enhances both diagnostic accuracy and confidence calibration compared to the full or equal responsibility models. Conversely, the equal responsibility framework, while appearing collaborative, was associated with poorer confidence calibration, a pattern that may be consistent with responsibility diffusion.

### Interpretation and Implications

Our findings align with and extend the existing literature on automation bias and the responsibility gap in health care. Some previous research has highlighted that clinicians may overrely on automated advice, particularly when liability is poorly defined. While some scholars have argued that placing full liability on clinicians is necessary to ensure safety, our data suggest that this sole accountability model (full responsibility) does not significantly improve diagnostic accuracy or calibration compared to the control. This contradicts the traditional “captain of the ship” legal doctrine, suggesting that liability pressure alone may be insufficient to overcome cognitive biases [15].

Our observation regarding the equal responsibility group may be consistent with research in social psychology concerning social loafing and responsibility diffusion. When accountability is shared equally, clinicians may distribute part of the cognitive burden to the AI, which may help explain the observed poorer confidence calibration [16]. However, this mechanism was not directly measured in the present study. The superior performance of the dynamic responsibility model suggests that a human factors engineering approach that prompts clinicians at the moment of decision conflict may support diagnostic verification more effectively than static pretask disclosures [17].

For clinical governance and the design of health IT systems, this study offers potential implications. Current regulatory frameworks often treat responsibility as a static legal assignment. Our results suggest that AI-assisted workflows could consider incorporating accountability by design [18]. By embedding responsibility cues within the decision-making interface, specifically when the clinician and the AI disagree, system designers may help reduce the risk of automation bias and promote more rigorous cross-checking behavior. For policymakers, this suggests that the distribution of liability may not be appropriately conceptualized as a fixed binary (human vs machine) but as a dynamic framework that encourages active human oversight.

### Limitations

Several limitations should be acknowledged. First, participants were aware of the responsibility structure to which they were assigned, which may have influenced their diagnostic revision behavior. Second, the vignette-based online simulation used in this study could not fully reproduce the decision pressures encountered in real-world clinical settings, which may limit the generalizability of the findings to clinical practice. Third, perceived responsibility and responsibility diffusion were not directly measured. Accordingly, responsibility diffusion should be interpreted as a plausible explanation rather than an empirically established causal mechanism.

Future research could measure perceived responsibility and related constructs to examine whether these constructs help explain the relationship between responsibility allocation structures and diagnostic performance. Building on this, studies should further evaluate the effects of different responsibility allocation structures in settings involving more realistic decision pressures and should investigate the long-term effects of dynamic responsibility structures. It is possible that alert fatigue could diminish the effectiveness of dynamic cues over time. Additionally, studies should explore how different levels of AI transparency interact with responsibility structures. For instance, would a black-box AI require more aggressive responsibility framing than a transparent one to achieve a comparable level of clinician vigilance? Exploring these interactions may contribute to the development of robust safety standards for AI integration in medicine.

### Conclusions

In summary, this study provides causal evidence that responsibility allocation structures can shape clinicians’ performance in AI-assisted clinical decision-making. The safe and effective use of clinical AI depends not only on algorithmic performance but also on whether human responsibility is consistently reinforced through organizational arrangements and clinical workflows. Compared with static responsibility arrangements, dynamic responsibility cues delivered at key decision points may better support clinicians’ active oversight and encourage more careful scrutiny of AI recommendations. These findings suggest that clinical AI governance should address not only technical reliability but also responsibility mechanisms and the risks associated with human-AI collaboration, thereby supporting safer and more trustworthy clinical use.

## Supplementary material

10.2196/97588 Checklist 1 CONSORT checklist.
